# Supplementary material for: Saccharomyces boulardii (CNCM I-745) improves intestinal damage in sepsis by remodeling intestinal flora structure
Source: Front Cell Infect Microbiol. 2025 Jul 31;15:1602792. doi: 10.3389/fcimb.2025.1602792 (PMC12350329; doi:10.3389/fcimb.2025.1602792)
Supplement: Supplementary file 1 [file DataSheet1.zip › summary/summary/7_Differential_analysis/2_LEfSe_result/Group_CW4_vs_CW0_0.05_3.0/LEfSe_Group_CW4_vs_CW0.abund.pdf]

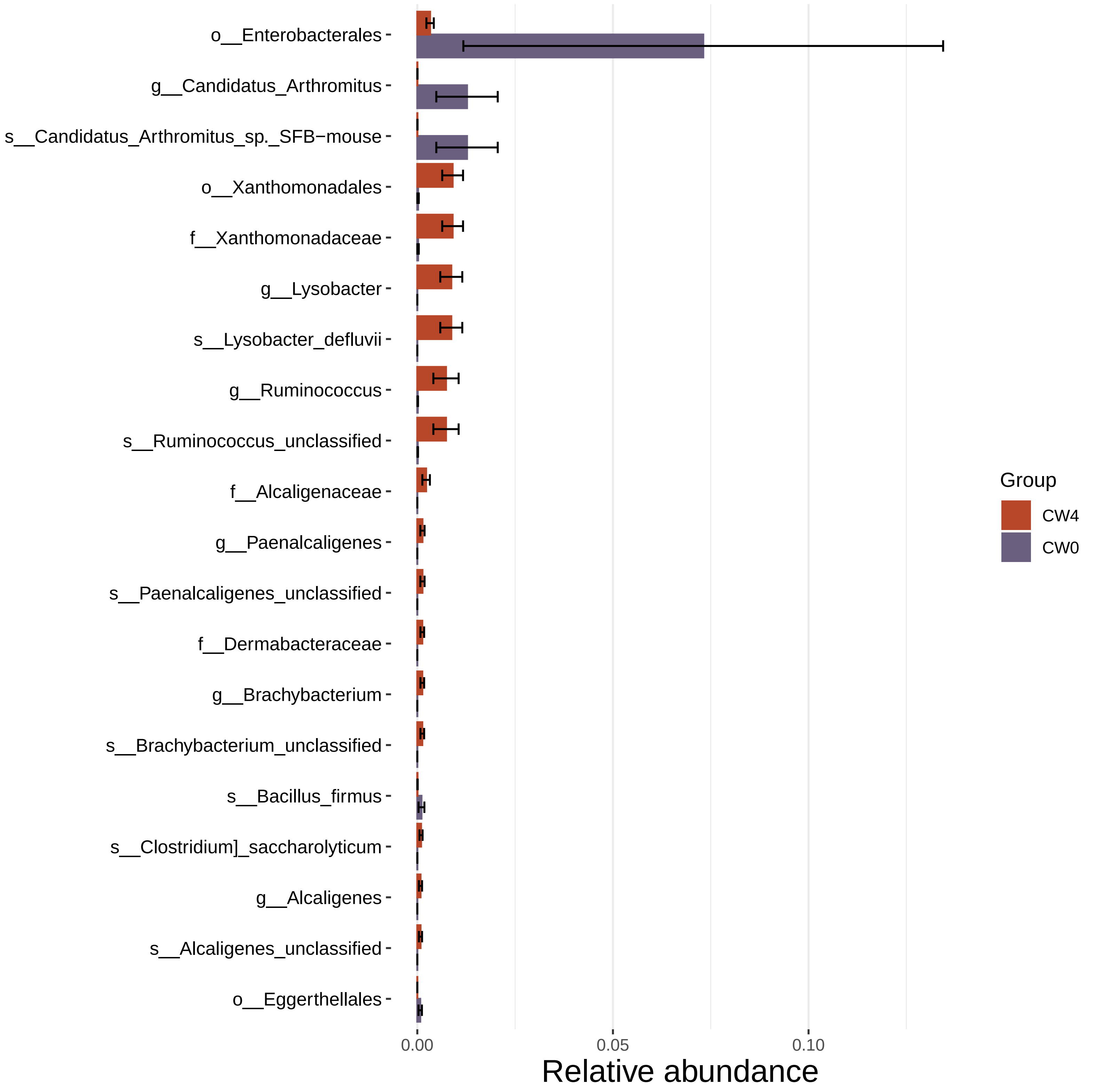
o__Enterobacterales
g__Candidatus_Arthromitus
s__Candidatus_Arthromitus_sp._SFB−mouse
o__Xanthomonadales
f__Xanthomonadaceae
g__Lysobacter
s__Lysobacter_defluvii
g__Ruminococcus
s__Ruminococcus_unclassified
f__Alcaligenaceae
g__Paenalcaligenes
s__Paenalcaligenes_unclassified
f__Dermabacteraceae
g__Brachybacterium
s__Brachybacterium_unclassified
s__Bacillus_firmus
s__Clostridium]_saccharolyticum
g__Alcaligenes
s__Alcaligenes_unclassified
o__Eggerthellales
0.00
0.05
0.10
Relative abundance
Group
CW4
CW0
